# Supplementary material for: Febrile Neutropenia Duration Is Associated with the Severity of Gut Microbiota Dysbiosis in Pediatric Allogeneic Hematopoietic Stem Cell Transplantation Recipients
Source: Cancers (Basel). 2022 Apr 12;14(8):1932. doi: 10.3390/cancers14081932 (PMC9026899; doi:10.3390/cancers14081932)
Supplement: Supplementary file 1 [file cancers-14-01932-s001.zip › cancers-1607127-supplementary/cancers-1607127-supplementary.pdf]

cancers

MDPI

Article

Febrile neutropenia duration is associated with the severity of gut microbiota dysbiosis in pediatric allogeneic hematopoietic stem cell transplantation recipients

Riccardo Masetti[1,2*], Federica D'Amico[2,3*], Daniele Zama[2,4], Davide Leardini[1†], Edoardo Muratore[1], Marek Ussowicz[5], Jowita Fraczkiewicz[5], Simone Cesaro[6], Giulia Caddeo[6], Vincenza Pezzella[6], Tamara Belotti[1], Francesca Gottardi[1], Piero Tartari[1], Patrizia Brigidi[2], Silvia Turroni[3*] and Arcangelo Prete[1*]

[1] Pediatric Oncology and Hematology "Lalla Seràgnoli", IRCCS Azienda Ospedaliero-Universitaria di Bologna, 40138 Bologna, Italy: riccardo.masetti5@unibo.it (R.M.), davide.leardini3@studio.unibo.it (D.L.), edoardo.muratore@studio.unibo.it (E.M.), tamara.belotti@aosp.bo.it (T.B.), francescag.ageop@aosp.bo.it (F. G.), piero.tartari@aosp.bo.it (P.T.), arcangelo.prete@aosp.bo.it (A.P.);

[2] Department of Medical and Surgical Sciences (DIMEC), University of Bologna, 40138 Bologna, Italy: federica.damico8@unibo.it (F.D.), patrizia.brigidi@unibo.it (P.B.);

[3] Department of Pharmacy and Biotechnology (FABIT), University of Bologna, 40126 Bologna, Italy: silvia.turroni@unibo.it (S.T.);

[4] Pediatric Emergency Unit, IRCCS Azienda Ospedaliero-Universitaria di Bologna, 40138 Bologna, Italy: daniele.zama2@unibo.it (D.Z.);

[5] Department and Clinic of Pediatric Oncology, Hematology and Bone Marrow Transplantation, Wroclaw Medical University, 50-556 Wrocław, Poland: ussowicz@o2.pl (M.U.), jowita.fraczkiewicz@umed.wroc.pl (J.F.);

[6] Department of Pediatric Hematology Oncology, Azienda Ospedaliera Universitaria Integrata, 37126 Verona, Italy: simone.cesaro@aovr.veneto.it (S.C.), giulia.caddeo@aovr.veneto.it (G.C.), vincenza.pezzella@aovr.veneto.it (V.P.)

[†] Correspondence: davide.leardini3@studio.unibo.it; Tel.: +39 051 214 4665, Pediatric Oncology and Hematology "Lalla Seràgnoli", IRCCS Azienda Ospedaliero-Universitaria di Bologna, Via Giuseppe Massarenti 11, 40138 Bologna, Italy;

* R.M. and F.D. contributed equally to this work as co-first authors. S.T. and A.P. contributed equally to this work as co-last authors.

Supplementary Table S1: Detailed summary of infections in the two patient groups. Each isolated pathogen is categorized following the Common Commensal tab of the NHSN Organisms List: CC - Organisms categorized as Common Commensals, MBI - Organisms categorized for Mucosal Barrier Injury, UTI - Organisms categorized for Urinary Tract Infection

Code	Group	BSI	Bacterial pathogen	Fungi	Viruses	Viral pathogen
BO_FN_01	>3 days	Yes	*Micrococcus luteus* (CC,UTI)	No	No	
BO_FN_02	>3 days	Yes	*Streptococcus oralis* (CC,MBI,UTI)	No	Yes	HHV6
BO_FN_03	>3 days	No		Yes	Yes	CMV
BO_FN_04	≤3 days	No		No	No	
BO_FN_05	>3 days	No		No	No	
BO_FN_06	≤3 days	No		No	No	
BO_FN_11	≤3 days	No		No	Yes	CMV
BO_FN_13	>3 days	No		No	Yes	BK, Adenovirus
BO_FN_14	>3 days	No		No	Yes	CMV
BO_FN_15	≤3 days	No		No	Yes	CMV
BO_FN_16	>3 days	Yes	*Streptococcus dysgalactiae* (UTI)	No	Yes	BK
BO_FN_17	>3 days	Yes	MSSA (UTI)	No	Yes	CMV
BR_FN_01	≤3 days	No		No	No	
BR_FN_02	≤3 days	No		No	No	
BR_FN_03	≤3 days	No		No	No	
BR_FN_04	≤3 days	No		No	No	
BR_FN_05	>3 days	No		No	No	
BR_FN_07	≤3 days	Yes	*Staphylocococcus hominis* MRS (CC,UTI)	No	No	
BR_FN_08	≤3 days	Yes	*Proteus mirabilis* (MBI,UTI)	No	No	
BR_FN_11	≤3 days	No		No	No	
BR_FN_12	≤3 days	No		No	No	
BR_FN_13	>3 days	No		Yes	No	
BR_FN_14	≤3 days	No		No	No	
BR_FN_15	>3 days	No		Yes	No	
BR_FN_17	>3 days	No		Yes	No	
VR_FN_01	≤3 days	No		No	No	
VR_FN_02	≤3 days	No		No	No	
VR_FN_03	≤3 days	No		No	No	
VR_FN_05	>3 days	No		No	Yes	CMV
VR_FN_07	≤3 days	No		No	No	
VR_FN_08	>3 days	No		No	No	
VR_FN_09	≤3 days	No		No	No	
VR_FN_10	≤3 days	No		No	No	
VR_FN_11	>3 days	No		Yes	No	
VR_FN_12	≤3 days	Yes	*Pseudomonas aeruginosa* (UTI)	No	No	
VR_FN_13	≤3 days	No		No	No	
VR_FN_14	>3 days	No		No	Yes	CMV

Supplementary Table S2: Detailed summary of administered antibiotics.

Code	Antibiotic prophylaxis	3° gen. cephalosporin	4° gen. cephalosporin	5° gen. cephalosporin	Amikacin	Carbapenem	Glycopeptide	Penicillin	Daptomycin	Linezolid	Metronidazole	Colistin	Clindamycin	Days of antibiotic before day +30	Days of neutropenic fever before day +30
BO_FN_01	no					■	■	■	■					18	16
BO_FN_02	no	■					■							9	7
BO_FN_03	no	■		■		■		■		■				28	20
BO_FN_04	no	■				■		■	■					5	3
BO_FN_05	no	■						■						25	10
BO_FN_06	no	■												8	3
BO_FN_11	no	■												9	1
BO_FN_13	no	■				■			■					21	13
BO_FN_14	no					■	■	■	■	■				20	16
BO_FN_15	no	■				■		■						21	3
BO_FN_16	no	■				■	■			■				20	5
BO_FN_17	no	■						■						20	5
BR_FN_01	no		■		■									30	2
BR_FN_02	Ciprofloxacin		■		■			■						16	2
BR_FN_03	Ciprofloxacin		■		■									16	3
BR_FN_04	Ciprofloxacin					■	■					■		23	3
BR_FN_05	no		■				■							12	4
BR_FN_07	no						■							7	3
BR_FN_08	no		■			■	■							8	3
BR_FN_11	no				■		■							10	2
BR_FN_12	Colistin		■		■	■		■						28	3
BR_FN_13	no					■				■		■		10	5
BR_FN_14	Ciprofloxacin						■							20	1
BR_FN_15	Ciprofloxacin, Rifaximin					■	■							30	12
BR_FN_17	Ciprofloxacin					■							■	10	5
VR_FN_01	Ampicillin					■								20	0
VR_FN_02	Ampicillin				■	■	■							26	0
VR_FN_03	Amoxicillin, Piperacillin-tazobactam				■	■								26	1
VR_FN_05	Ampicillin					■		■						24	9
VR_FN_07	Piperacillina-tazobactam													0	0
VR_FN_08	Amoxicillin				■	■	■							26	12
VR_FN_09	Ampicillin	■			■	■	■				■			13	1
VR_FN_10	Piperacillin-tazobactam						■				■			17	1
VR_FN_11	Amoxicillin		■		■		■				■			32	5
VR_FN_12	Amoxicillin	■			■	■	■							23	1
VR_FN_13	Amoxicillin				■	■	■				■			19	2
VR_FN_14	Ampicillin	■			■		■							24	27

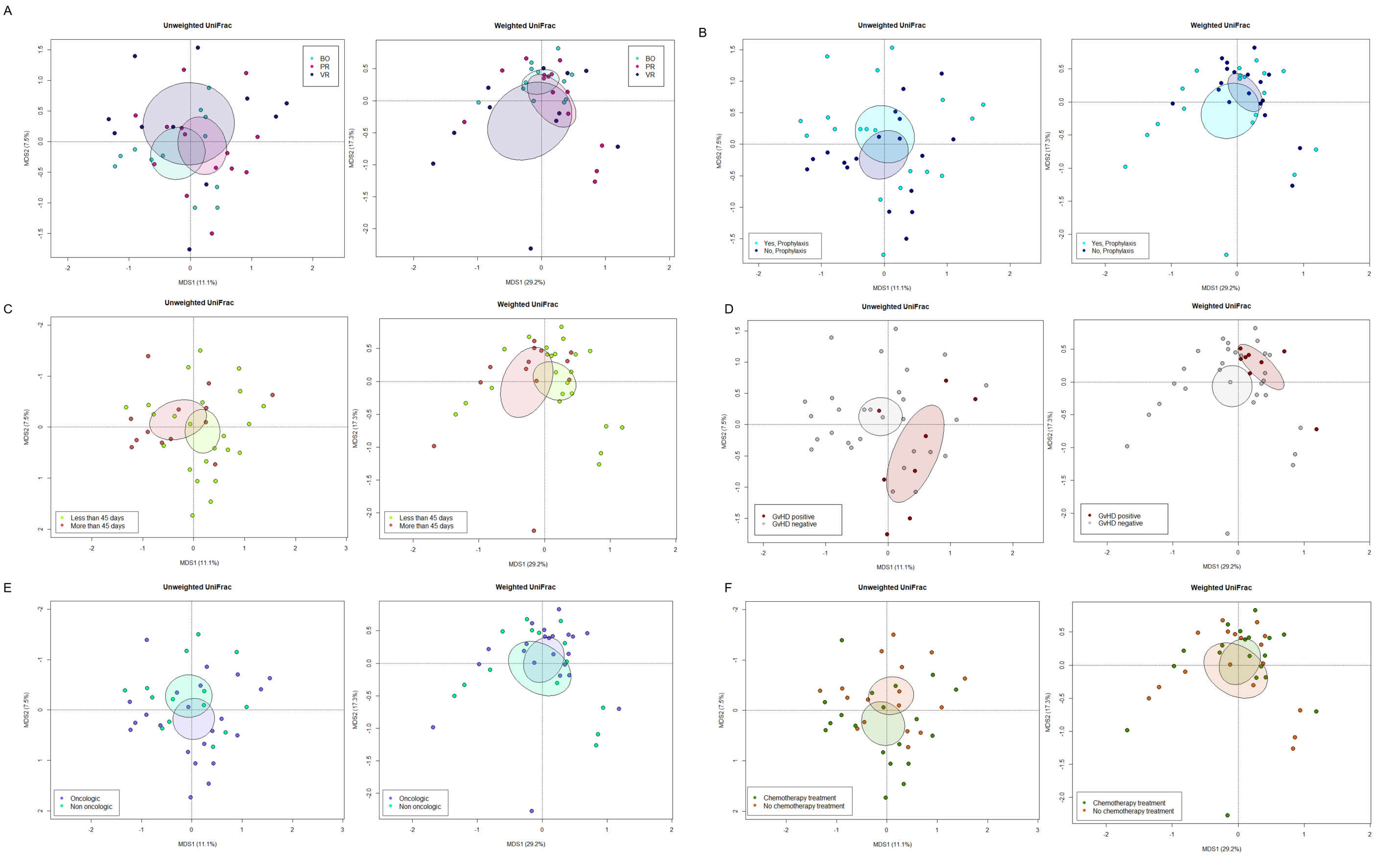

Supplementary Figure S1. Baseline gut microbiota diversity in pediatric allo-HSCT patients who developed febrile neutropenia. PCoA based on unweighted (left) and weighted (right) UniFrac distances between the gut microbiota profiles of samples collected before transplant and stratified by transplant center (Bologna, BO vs. Verona, VR vs. Wroclaw, PR, A), antibiotic prophylaxis (yes vs. no, B), repeated antibiotic therapies (less vs. more than 45 days before sampling, C), GvHD development (positive vs. negative, D), underlying disease (oncologic vs. non-oncologic, E) and previous chemotherapy cycles (yes vs. no, F). Ellipses include 95% confidence area based on the standard error of the weighted average of sample coordinates. No significant separation between groups was found (permutation test with pseudo-F ratio, $p \geq 0.08$). The 45-day cut-off for previous antibiotic therapies was chosen based on Palleja et al., 2018.

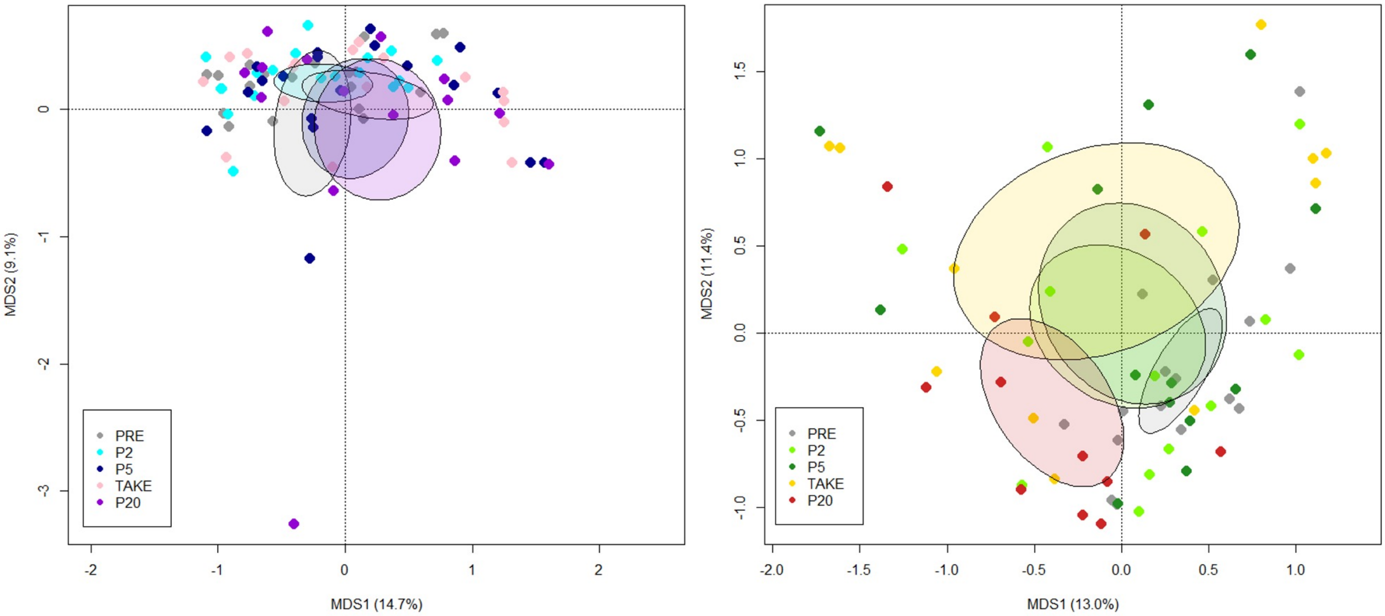

Supplementary Figure S2. Beta diversity of the gut microbiota in pediatric allo-HSCT patients with different duration of febrile neutropenia. PCoA based on unweighted UniFrac distances between the gut microbiota profiles of samples collected before transplant (PRE), at the onset of neutropenia (day -2/+2; P2), at the onset of fever (day +4/+5; P5), at engraftment (TAKE) and after engraftment (day +20/+30; P20), in pediatric allo-HSCT patients with less (left) and more (right) than three days of fever. Ellipses include 95%confidence area based on the standard error of the weighted average of sample coordinates. Significant separation among groups was found only for patients with longer fever duration (permutation test with pseudo-F ratio, $p = 0.05$).

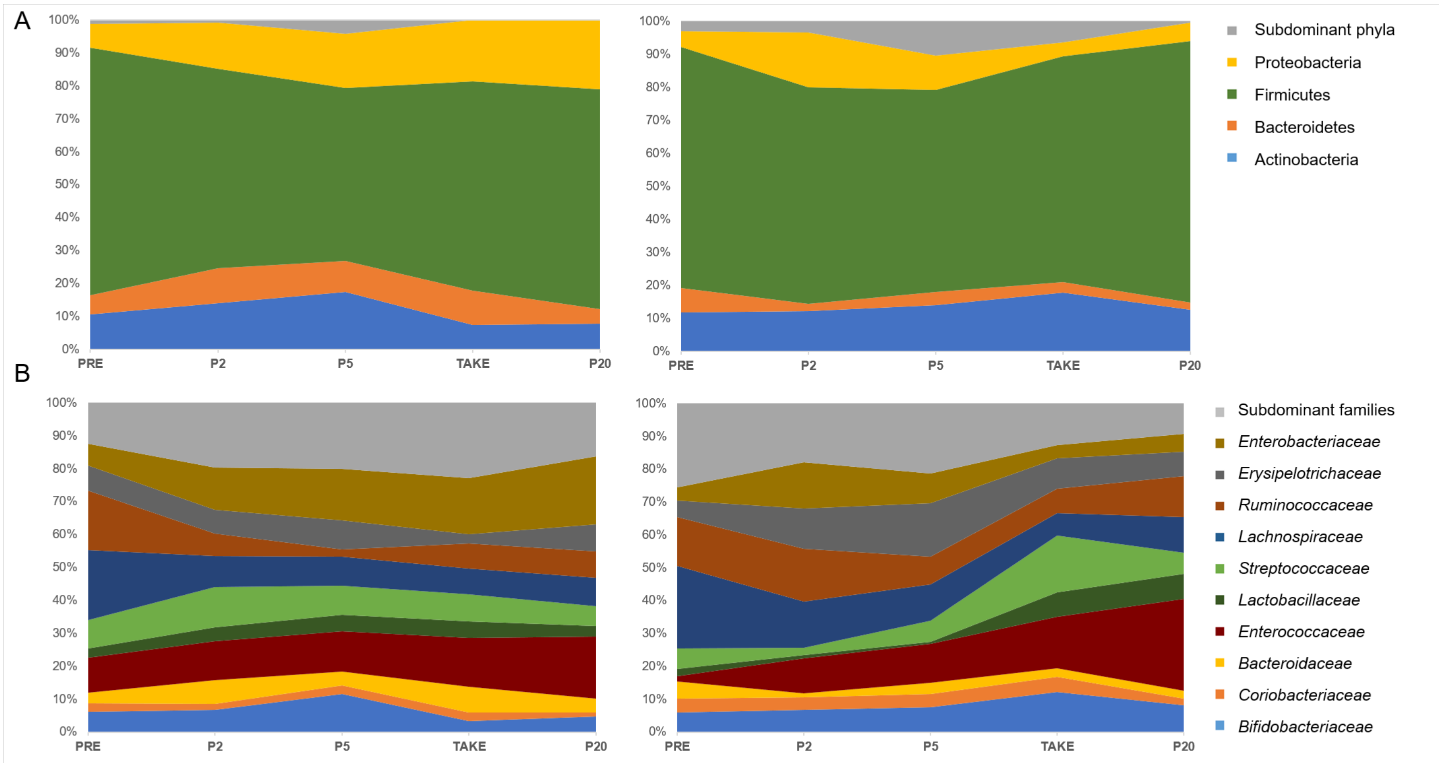

Supplementary Figure S3. Gut microbiota trajectory in pediatric allo-HSCT patients with different duration of febrile neutropenia. Area plots representing the relative abundance of the major phyla (A) and families (B) in the GM of patients with less (left) and more (right) than three days of fever. Only taxa with mean relative abundance > 20% in at least five samples of the total dataset are shown. PRE, before transplant; P2, at the onset of neutropenia (day -2/+2); P5, at the onset of fever (day +4/+5); TAKE, at engraftment; P20, after engraftment (day +20/+30).

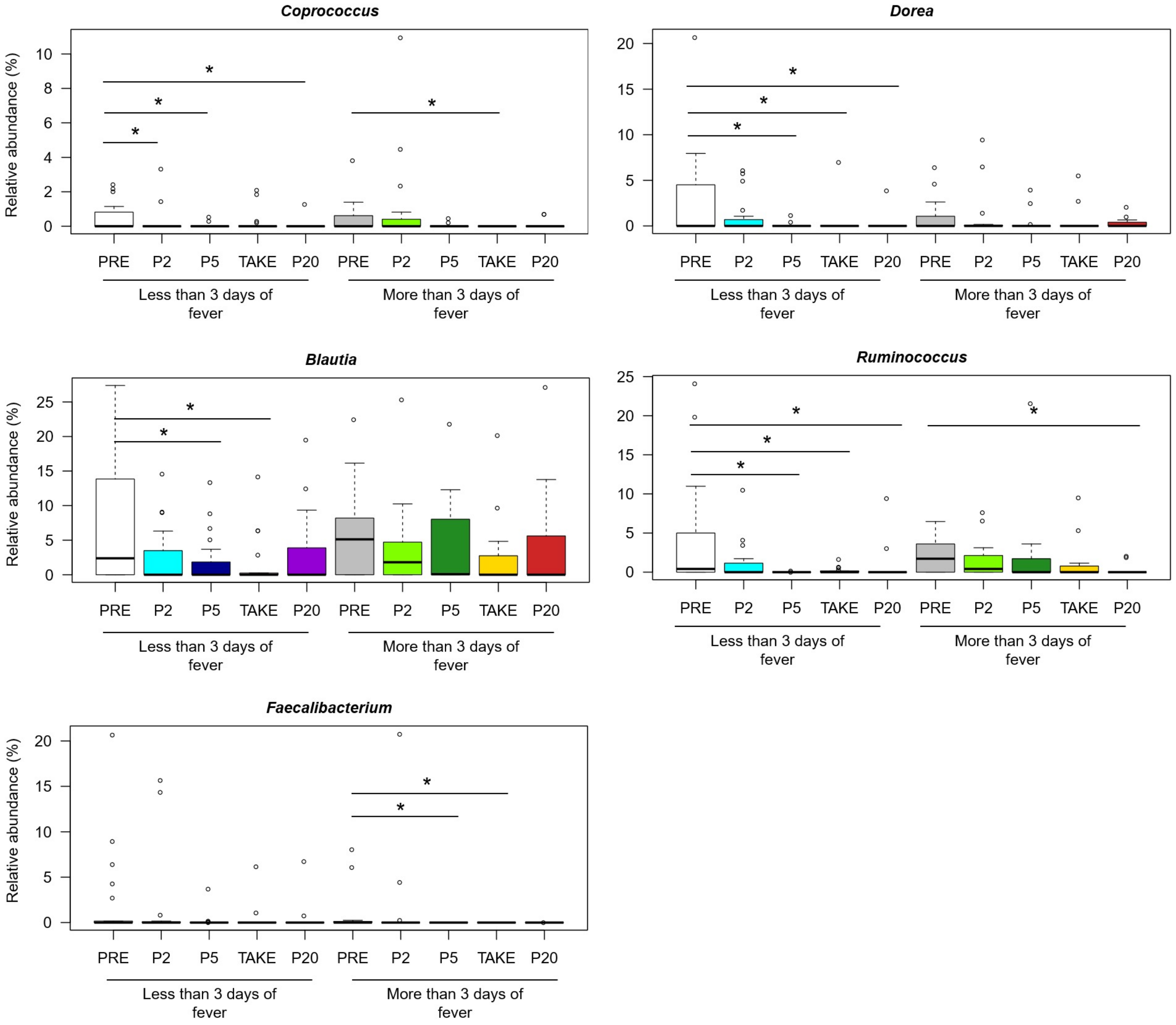

Supplementary Figure S4. Dynamics of typical health-associated taxa in pediatric allo-HSCT patients with different duration of febrile neutropenia. Boxplots showing the relative abundance distribution of typical health-associated genera significantly differentially represented over time in patients with different duration of fever (*i.e.*, less vs. more than three days of fever). Wilcoxon test, * for $p < 0.05$.

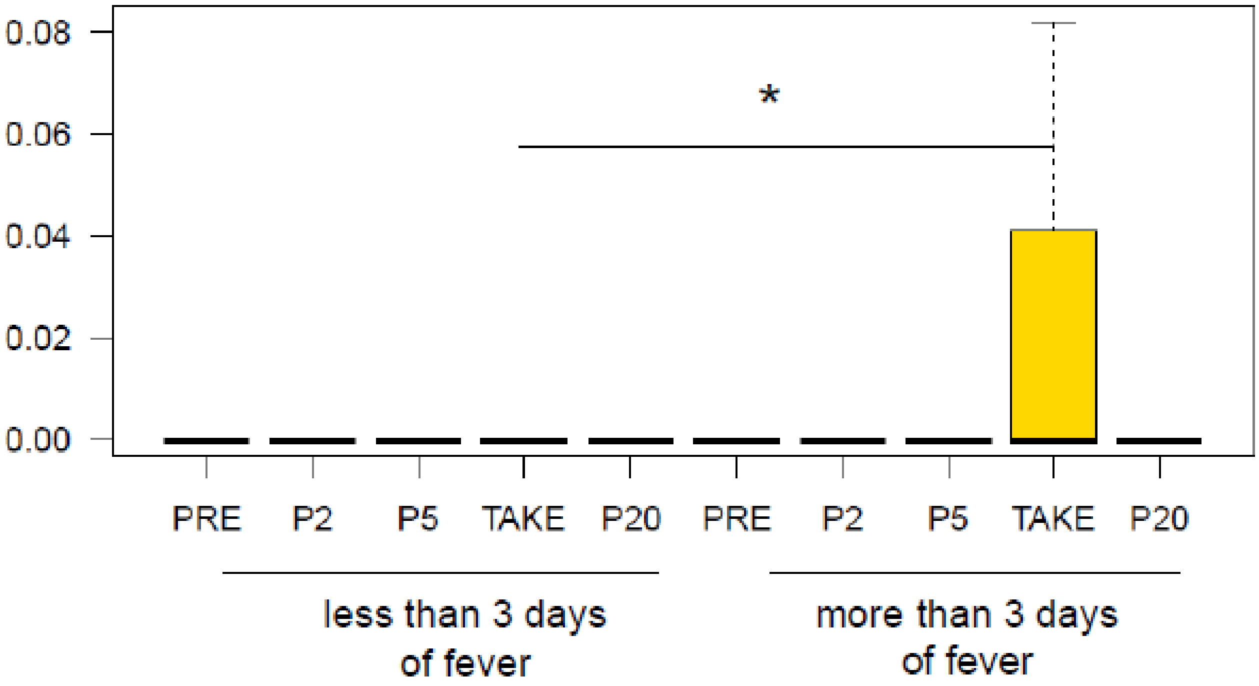

Supplementary Figure S5. Boxplots showing the relative abundance distribution of *Akkermansia* in patients with different duration of febrile neutropenia (less vs. more than three days). PRE, before transplant; P2, at the onset of neutropenia (day -2/+2); P5, at the onset of fever (day +4/+5); TAKE, at engraftment; P20, after engraftment (day +20/+30). Wilcoxon test, * for $p < 0.05$.
